# Health Care Professionals’ Clinical Perspectives and Acceptance of a Blood Glucose Meter and Mobile App Featuring a Dynamic Color Range Indicator and Blood Sugar Mentor: Online Evaluation in Seven Countries

**DOI:** 10.2196/13847

**Published:** 2019-07-03

**Authors:** Mike Grady, Usha Venugopal, Katia Robert, Graham Hurrell, Oliver Schnell

**Affiliations:** 1 LifeScan Scotland Inverness United Kingdom; 2 LifeScan Europe GmbH Zug Switzerland; 3 LifeScan Global Corporation Wayne, PA United States; 4 Forschergruppe Diabetes eV München-Neuherberg Germany

**Keywords:** ColorSure Dynamic Range Indicator, Blood Sugar Mentor, mobile app, blood glucose meter, self-monitoring of blood glucose, health care professionals

## Abstract

**Background:**

Despite many new therapies and technologies becoming available in the last decade, people with diabetes continue to struggle to achieve good glycemic control. Innovative and affordable solutions are needed to support health care professionals (HCPs) to improve patient outcomes.

**Objective:**

To gather current self-management perceptions of HCPs in seven countries and investigate HCP satisfaction with a new glucose meter and mobile app featuring a dynamic color range indicator and a blood sugar mentor.

**Methods:**

A total of 355 HCPs, including 142 endocrinologists (40.0%), 108 primary care physicians (30.4%), and 105 diabetes nurses (29.6%), were recruited from the United Kingdom (n=50), France (n=50), Germany (n=50), India (n=54), Algeria (50), Canada (n=51), and the United States (n=50). HCPs experienced the OneTouch Verio Reflect glucose meter and the OneTouch Reveal mobile app online from their own office computers using interactive demonstrations via webpages and multiple animations. After providing demographic and clinical practice insights, HCPs responded to statements about the utility of the system.

**Results:**

Concerning current practice, 83.1% (295/355) of HCPs agreed that poor numeracy or health literacy was a barrier for their patients. A total of 85.9% (305/355) and 92.1% (327/355) of HCPs responded that type 2 diabetes (T2D) and type 1 diabetes (T1D) patients were aware of what represented a low, in-range, or high blood glucose result. Only 62.0% (220/355) felt current glucose meters made it easy for patients to understand if results were in range. A total of 50.1% (178/355) and 78.0% (277/355) of HCPs were confident that T1D and T2D patients took action for low or high results. A total of 87.0% (309/355) agreed that the ColorSure Dynamic Range Indicator could help them teach patients how to interpret results and 88.7% (315/355) agreed it made them more aware of hyper- and hypoglycemic results so they could take action. A total of 83.7% (297/355) of HCPs agreed that the Blood Sugar Mentor feature gave personalized guidance, insight, and encouragement so patients could take action. A total of 82.8% (294/355) of HCPs also agreed that the Blood Sugar Mentor provided real-time guidance to reinforce the goals HCPs had set so patients could take steps to manage their diabetes between office visits. After experiencing the full system, 85.9% (305/355) of HCPs agreed it was beneficial for patients with lower numeracy or health literacy; 96.1% (341/355) agreed that it helped patients understand when results were low, in range, or high; and 91.0% (323/355) agreed that the way it displayed diabetes information would make patients more inclined to act upon results. A total of 89.0% (316/355) of HCPs agreed that it would be helpful for agreeing upon appropriate in-range goals for their patients for their next clinic visit.

**Conclusions:**

This multi-country online study provides evidence that HCPs were highly satisfied with the OneTouch Verio Reflect meter and the OneTouch Reveal mobile app. Each of these use color-coded information and the Blood Sugar Mentor feature to assist patients with interpreting, analyzing, and acting upon their blood glucose results, which is particularly beneficial to keep patients on track between scheduled office visits.

## Introduction

Despite many new technologies and medications to treat diabetes during the past decade, the proportion of patients in the United States achieving a glycated hemoglobin (HbA_1c_) target of less than 7.0% has been fairly constant from 2003 to 2010, remaining at just over 50% [1]. A further analysis confirmed that those achieving an HbA_1c_ of less than 7.0% only slightly declined from 52.2% to 50.9% by 2014, and the proportion in poor control (ie, HbA_1c_ >9.0%) actually worsened from 12.6% to 15.5% from 2007 to 2014 [2]. Even among patients with type 1 diabetes (T1D) who are part of the so called “T1D exchange” dataset in specialty diabetes centers in the United States, mean HbA_1c_ worsened from 7.8% in 2010-2012 to 8.4% in 2016-2018, despite continuous glucose monitoring usage increasing from 7% to 30% in this population [3]. These findings are concerning and would seem to indicate that other factors are preventing patients from taking full advantage of self-monitoring or continuous glucose monitoring technologies, which may include patients simply struggling to interpret and act upon information from these technologies. In fact, the latest American Diabetes Association guidelines suggest that optimal use of self-monitoring technologies requires proper review and interpretation of the data by both the patient and the provider to ensure that data are used in an effective and timely manner; the guidelines also suggest that patients should be taught how to use their self-monitoring data to adjust food intake, exercise, or medications to achieve specific goals [4]. Appropriate education addressing how to interpret self-monitoring of blood glucose information and how to respond to *out-of-range* results have been identified as important requirements for useful self-monitoring of blood glucose practice [5]. However, a survey of 886 people showed that approximately 50% of insulin and non-insulin-using patients with type 2 diabetes (T2D) regularly took no action for out-of-range readings, low or high, with any self-care adjustments [6]. Disparities in literacy and low numeracy in patients in various countries could impede efforts to support patients who struggle to comprehend self-care guidance or use the self-monitoring technologies provided by health care professionals (HCPs). For example, low diabetes-related numeracy skills are associated with fewer self-management behaviors [7], and poor numeracy is also associated with suboptimal glycemic control in patients with T2D [8] and T1D [9]. We previously reported that glucose meters utilizing color range indicators improved the ability of patients to interpret glucose results [10], improved decision making in terms of taking action [11], and improved glycemic control when patients switched from other glucose meters [12-14]. In this study, we solicited feedback from HCPs in seven countries to explore current clinical practice issues with respect to self-monitoring and to determine how the OneTouch Verio Reflect meter, as well as the OneTouch Reveal mobile app, with ColorSure Dynamic Range Indicator and Blood Sugar Mentor (BSM) features might provide benefits for patients in these countries.

## Methods

### Materials

**Figure 1:**
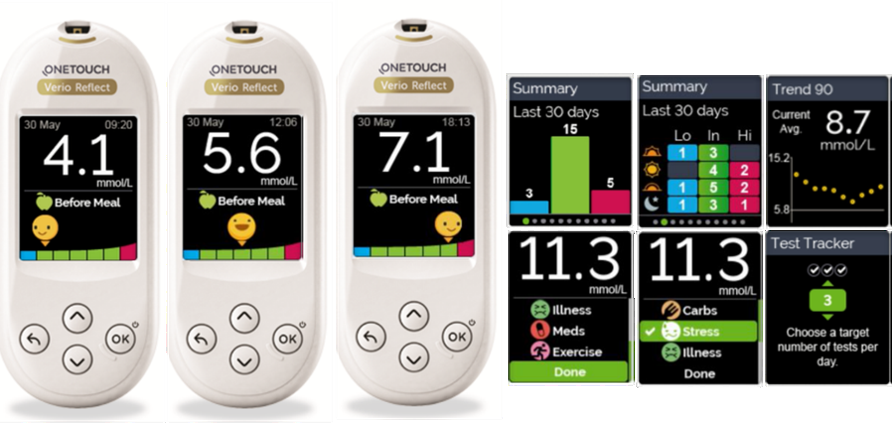
OneTouch Verio Reflect meter showing a selection of screens.

OneTouch Verio Reflect (LifeScan) is a blood glucose meter intended for self-testing by people with diabetes. The meter automatically analyzes high and low glucose patterns, tracks trends, and provides on-meter guidance messages to help patients understand and manage their glucose levels and help them detect excursions above or below a desired glucose range. The meter has a ColorSure Dynamic Range Indicator to show when a result is low (blue), in range (green), or high (red) using seven color-coded segments. The patient or HCP can personalize the ranges of this feature and choose between an arrow or emoji character to indicate which colored segment relates to the current reading. The meter has additional features over basic glucose meters, including a test goal tracker; advanced tagging of results with various event symbols, such as carbohydrates, stress, illness medication, or exercise; a color grid of the last 30 days of results (ie, time of day/glucose range); and provides an on-meter trend graph of average glucose values (see Figure 1).

**Figure 2:**
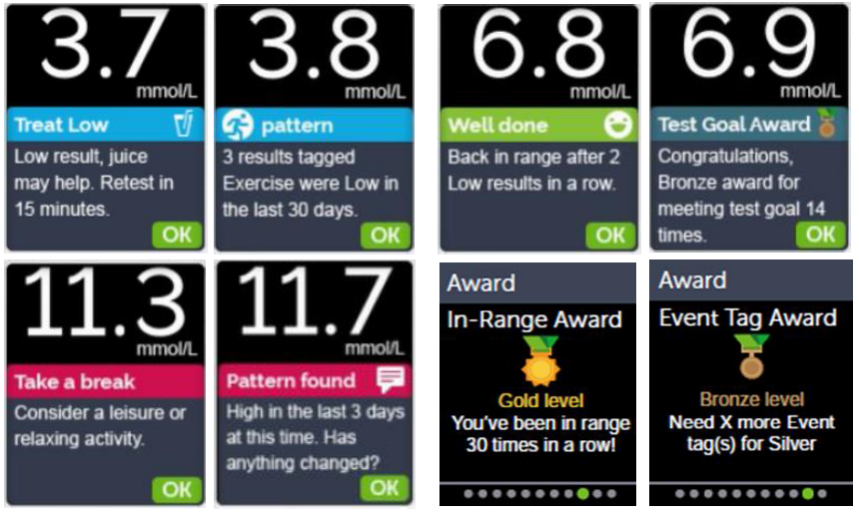
Selected Blood Sugar Mentor screens on the OneTouch Verio Reflect meter.

Furthermore, the on-meter Blood Sugar Mentor feature provides a variety of mentor tips, pattern messages, and awards when certain testing achievements or glycemic goals are met (see Figure 2), such as the following:

1. Mentor Tips are provided when results are consistently in range or are currently trending low or high.
2. Pattern Messages are provided when the meter identifies a pattern of glucose results that fall outside the high and low range limits the user sets in the meter.
3. Awards are earned when award criteria are met, such as meeting the daily test goal.

**Figure 3:**
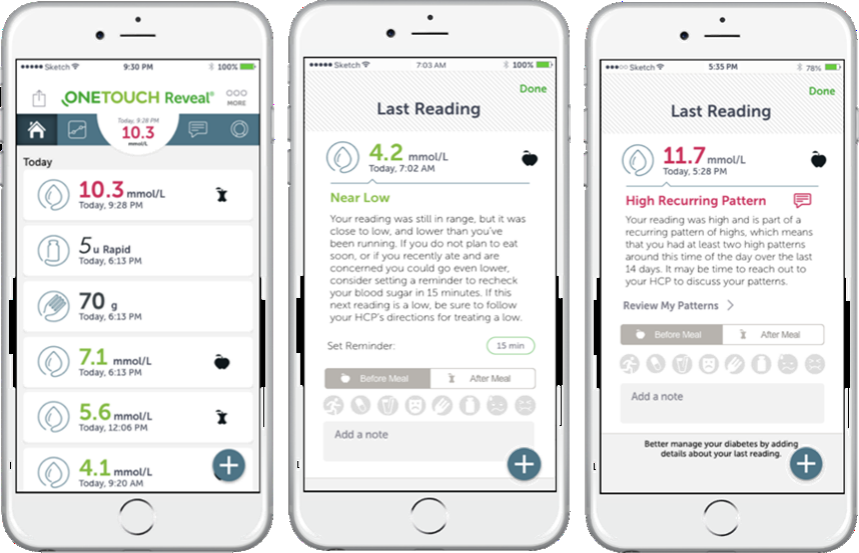
Selected Blood Sugar Mentor messages on the OneTouch Reveal app.

The Verio Reflect meter has Bluetooth capability to allow transfer of blood glucose readings and event tagging information to the OneTouch Reveal app (eg, on a mobile phone or tablet), which has additional insight and trending features and provides HCPs and patients with the ability to share information during and between clinic visits (see Figure 3).

### Procedure

This multi-country online survey study was conducted by individual HCPs from institutions and clinical practices within each country. Webpages containing text information, product and feature images, and links to short animations describing meter and app features were provided to the HCPs. A total of 355 HCPs from seven countries—the United Kingdom, France, Germany, India, Algeria, Canada, and the United States—were recruited and included endocrinologists, primary care physicians (PCPs), and diabetes nurses. The sampling strategy dictated a target of 20 endocrinologists, 15 physicians, and 15 diabetes nurses per country to ensure we had representative views from different HCPs. Online inclusion criteria questions precluded HCPs who did not routinely treat at least 10 T1D or T2D patients per week or who did not have at least 15% of their patient population currently on insulin therapy. Before the online experience, all HCPs provided demographic and clinical practice metrics with respect to the patients they routinely advised or treated.

HCPs were asked eight clinical practice questions to determine the confidence they had in the ability of their current patients with type 1 and type 2 diabetes to interpret or act upon blood glucose data. Participating HCPs were then presented with an online experience sharing identical capability, functionality, and navigation as the intended meter and app products. The meter and app animations were preloaded with representative glucose results, messages, guidance, or information that provided examples of the meter screens that would appear whenever HCPs or patients review information. The HCPs interacted online with a series of webpages displaying both text and visuals of the meter and app, with embedded links on the selected webpages allowing HCPs to watch short product animations. At various points during these activities, 24 survey questions were presented to assess the HCPs’ opinions of the value of various functions and features of the meter and app. After completing online activities, HCPs were asked four clinical practice-based questions pertaining to the value of the meter and app for supporting patients in future with diabetes self-management.

### Statistical Analyses

Continuous demographic variables were described as median and range or mean and standard deviation. Categorical demographic variables were described as percentages within categories and are presented with both numerators and denominators. HCP responses to survey statements were recorded using a 5-point Likert scale with a favorable response (4 or 5) being met if the lower 95% one-sided confidence limit for the percentage of participants providing a favorable response per item was greater than 50%.

## Results

### Health Care Professionals’ Demographic and Clinical Practice Information

A total of 355 HCPs took part in the study from seven countries: the United Kingdom (n=50), France (n=50), Germany (n=50), India (n=54), Algeria (n=50), Canada (n=51), and the United States (n=50). Out of the 355 HCPs, professional backgrounds included 142 endocrinologists (40.0%), 108 PCPs (30.4%), and 105 diabetes nurses (29.6%). Mean age across all seven countries was 48 years (SD 9) for endocrinologists, 49 years (SD 9) for PCPs, and 46 years (SD 9) for diabetes nurses. The proportion of patients with T1D and T2D, respectively, typically seen by each professional in routine clinical practice was 25% and 69% for endocrinologists, 16% and 80% for PCPs, and 27% and 67% for diabetes nurses (see Table 1).

**Table 1:** Health care professionals’ status and patient population information.

Patient population information		Health care professionals			
		Endocrinologists (n=142)	Primary care physicians (n=108)	Diabetes nurses (n=105)	Combined (N=355)
**Gender, n (%)**					
	Male	99 (69.7)	79 (73.1)	23 (21.9)	201 (56.6)
	Female	43 (30.3)	29 (26.9)	82 (78.1)	154 (43.4)
Age in years, mean (SD)		48 (9)	49 (9)	46 (9)	48 (10)
**Patient diabetes type, mean %^a^**					
	Type 1 diabetes	25	16	27	23
	Type 2 diabetes	69	80	67	72
	Other	6	4	6	5
**Patient-specific therapy, mean %^a^**					
	Diet and exercise	9	12	11	11
	Diabetes medications only	31	45	29	35
	Diabetes medications and insulin	28	24	27	26
	Insulin only (injections or pump)	22	14	23	20
	Insulin plus CGM^b^ or FGM^c^	13	9	14	12

^a^Percentages shown are estimates given by the health care professionals.

^b^CGM: continuous glucose monitor.

^c^FGM: flash glucose monitor.

### Health Care Professionals’ Clinical Practice Feedback on Patient Self-Care

**Figure 4:**
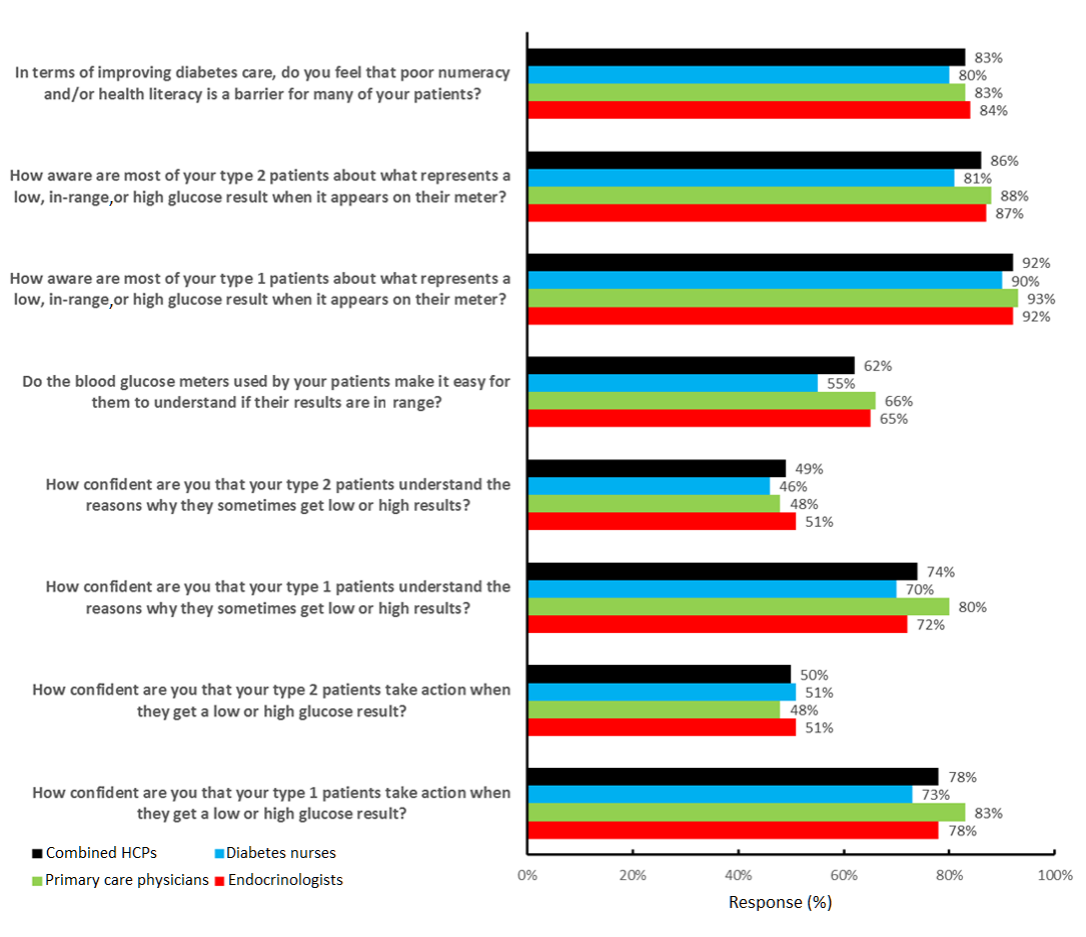
Health care professionals’ (HCPs) feedback regarding current patient self-care. Results shown are percentage favorable responses (strongly agree or agree; very confident or confident; very aware or aware) on a 5-point Likert scale ranging from 1 (strongly agree, very confident, or very aware) to 5 (strongly disagree, not at all confident, or not at all aware).

Of the HCPs who responded, 83.1% (295/355) confirmed that poor numeracy or health literacy was a barrier for many of their patients to improve their diabetes care. However, a high percentage of HCPs responded that their current patients were aware about what represents a low, in-range, or high glucose result when it appeared on their glucose meter, with 85.9% (305/355) in agreement for T2D patients and 92.1% (327/355) for T1D patients. Far fewer HCPs were convinced that the blood glucose meters used by their patients make it easy for them to understand if their results are in range, with only 62.0% (220/355) in agreement. In terms of confidence that their patients understood the reasons why they sometimes got low or high glucose results, the responses from HCPs were quite different in that only 49.0% (174/355) agreed that patients with T2D understood the reasons why, with a higher percentage of HCPs (263/355, 74.1%) agreeing that their T1D patients understood the reasons for low or high glucose results. This disparity was also evident when HCPs were asked how confident they were that their patients took action when they got a low or high glucose result, with only 50.1% (178/355) agreeing that patients with T2D took action, with a higher percentage of HCPs (277/355, 78.0%) agreeing that their T1D patients would take action for low or high glucose results (see Figure 4).

### Health Care Professionals’ Feedback During Online Experiences With the Meter and App

During the interactive online meter experience, 91.5% (130/142) of endocrinologists, 91.7% (99/108) of PCPs, and 89.5% (94/105) of nurses agreed that the ColorSure Dynamic Range Indicator could help patients understand when glucose results were near the high or low limits, so they could make adjustments before going out of range. A total of 86.6% (123/142) of endocrinologists, 88.9% (96/108) of PCPs, and 91.4% (96/105) of nurses also agreed that the ColorSure Dynamic Range Indicator could help patients be more aware of hyper- and hypoglycemic results so they could take action (see Table 2).

**Table 2:** Health care professionals’ responses to 24 survey statements about the OneTouch Verio Reflect meter and the OneTouch Reveal mobile app.

Survey statement	Favorable responses by health care professionals, n (%)^a^			
	Endocrinologists (n=142)	PCPs^b^ (n=108)	Nurses (n=105)	Combined (N=355)
The ColorSure Dynamic Range Indicator can help patients understand when glucose results are near high or low, so they can make adjustments before going out of range.	130 (91.5)	99 (91.7)	94 (89.5)	323 (91.0)
The ColorSure Dynamic Range Indicator will help patients be more aware of hyper and hypo results so they can take action.	123 (86.6)	96 (88.9)	96 (91.4)	315 (88.7)
I believe the meter will help patients reduce the number of hypoglycemic episodes they experience.	106 (74.6)	86 (79.6)	77 (73.3)	269 (75.8)
Patients can know if their actions are working with the enhanced blue, green, red ColorSure Dynamic Range Indicator that shows if they're in range, out of range, or near a high or low, and see that information directly on their smartphone.	125 (88.0)	99 (91.7)	95 (90.5)	319 (89.9)
The solution’s ColorSure technology could make it easy to teach my patients to interpret their blood glucose results.	122 (85.9)	94 (87.0)	93 (88.6)	309 (87.0)
The Blood Sugar Mentor will help patients understand the impact of food, activity, and medication on their glucose, so they can make adjustments to improve blood sugar control.	122 (85.9)	95 (88.0)	96 (91.4)	313 (88.2)
The Blood Sugar Mentor automatically identifies times when patients are likely to experience hyper and hypo events and alerts them, so they will be able to make changes to their daily routine.	123 (86.6)	88 (81.5)	94 (89.5)	305 (85.9)
The Blood Sugar Mentor analyses patterns, tracks trends, and could help guide patients toward better self-management and staying healthy.	122 (85.9)	95 (88.0)	95 (90.5)	312 (87.9)
The guidance, insights, and encouragement provided by the Blood Sugar Mentor will help patients take actions to manage their diabetes.	115 (81.0)	94 (87.0)	88 (83.8)	297 (83.7)
The real-time guidance that the Blood Sugar Mentor provides will help reinforce the goals you set, so patients can take steps to manage their diabetes between office visits.	115 (81.0)	90 (83.3)	89 (85.0)	294 (82.8)
I believe the meter will provide patients with greater understanding and guidance, so they can confidently make progress managing their blood sugar.	117 (82.4)	96 (88.9)	89 (84.8)	302 (85.1)
I believe the meter will help patients stay on top of their testing routine and control their glucose around meals, activities, and specific times of day.	106 (74.6)	94 (87.0)	86 (81.9)	286 (80.6)
I believe the meter will help patients manage their blood sugar more effectively than devices without a ColorSure Dynamic Range Indicator and Blood Sugar Mentor.	109 (76.8)	93 (86.1)	86 (81.9)	288 (81.1)
The solution's Blood Sugar Mentor will make it easy for patients to see and understand how their lifestyle choices impact their blood glucose levels, right on their meter and smartphone.	124 (87.3)	97 (89.8)	93 (88.6)	314 (88.5)
With the Blood Sugar Mentor, patients get personalized guidance, insight, and encouragement so they can take actions based on their current and previous results.	122 (85.9)	95 (88.0)	96 (91.4)	313 (88.2)
The solution's Blood Sugar Mentor could help patients be more proactive in managing their glucose levels.	116 (81.7)	88 (81.5)	94 (89.5)	298 (83.9)
The ongoing guidance provided by the solution’s Blood Sugar Mentor could help my struggling patients improve their understanding of diabetes management and take action.	115 (81.0)	97 (89.8)	88 (83.8)	300 (84.5)
With this solution, patients do not have to memorize facts, numbers, and actions, so they can confidently manage their diabetes.	114 (80.3)	90 (83.3)	83 (79.0)	287 (80.8)
The ColorSure Dynamic Range Indicator helps patients know when they are near hypo- and hyperglycemic levels and provides them with actions they could take to help avoid them.	119 (83.8)	90 (83.3)	94 (89.5)	303 (85.4)
The solution will make it easy to quickly see and assess my patients’ lifestyle and blood glucose data and help me to provide more personalized care.	103 (72.5)	91 (84.3)	90 (85.7)	284 (80.0)
The solution alerts patients when they are near hyper- or hypoglycemic levels and provides simple suggestions for corrective actions to help avoid them.	126 (88.7)	89 (82.4)	96 (91.4)	311 (87.6)
This solution provides patients with greater understanding and guidance in managing their blood sugar so they can confidently make progress toward their diabetes management goals.	121 (85.2)	95 (88.0)	90 (85.7)	305 (85.9)
The solution analyses and informs patients of blood glucose trends and potential causes, and provides personalized guidance patients can use to avoid hyper- and hypoglycemia.	120 (84.5)	93 (86.1)	92 (87.6)	305 (85.9)
The solution gives a better understanding of the underlying causes of patients’ blood glucose changes so they are better able to manage them.	112 (78.9)	90 (83.3)	81 (77.1)	283 (79.7)

^a^Results shown are favorable responses (*strongly agree* or *agree*) on a 5-point Likert scale ranging from 1 (*strongly agree*) to 5 (*strongly disagree*). All favorable responses met the acceptance criteria (ie, lower bound of 95% confidence limits >50%).

^b^PCP: primary care physician.

Minimizing the incidence of hypoglycemic events is a key part of diabetes care and a real concern for patients and HCPs. A total of 74.6% (106/142) of endocrinologists, 79.6% (86/108) of PCPs, and 73.3% (77/105) of nurses believed that this new meter will help patients reduce the number of hypoglycemic episodes they experience. This is an important endorsement and part of this advocacy may relate to the HCPs’ experience of the BSM features on the meter and app. A total of 86.6% (123/142) of endocrinologists, 81.5% (88/108) of PCPs, and 89.5% (94/105) of nurses agreed that the BSM automatically identified times when patients are likely to experience hyper- and hypoglycemic events and alerts them so they will be able to make changes to their daily routine. A total of 81.0% (115/142) of endocrinologists, 87.0% (94/108) of PCPs, and 83.8% (88/105) of nurses agreed that the guidance, insights, and encouragement provided by the BSM would also help their patients take action to manage their diabetes. Increasingly, it is what patients do themselves between visits that concerns HCPs. A total of 81.0% (115/142) of endocrinologists, 83.3% (90/108) of PCPs, and 84.8% (89/105) of nurses agreed that the real-time guidance that the BSM provides will help reinforce the goals that they set so patients can take steps to manage their diabetes between office visits. Overall, combining the attributes of the meter and the app together (ie, the solution), 88.7% (126/142) of endocrinologists, 82.4% (89/108) of PCPs, and 91.4% (96/105) of nurses agreed that the solution alerts patients when they are near hyper- or hypoglycemic levels and provides simple suggestions for corrective actions to help avoid them. In addition, 85.2% (121/142) of endocrinologists, 88.0% (95/108) of PCPs, and 85.7% (90/105) of nurses agreed that the solution provides patients with greater understanding and guidance in managing their blood sugar so they can confidently make progress toward their diabetes management goals.

### Health Care Professionals’ Clinical Practice Outlook for Patients Based on Meter and App Experiences

**Figure 5:**
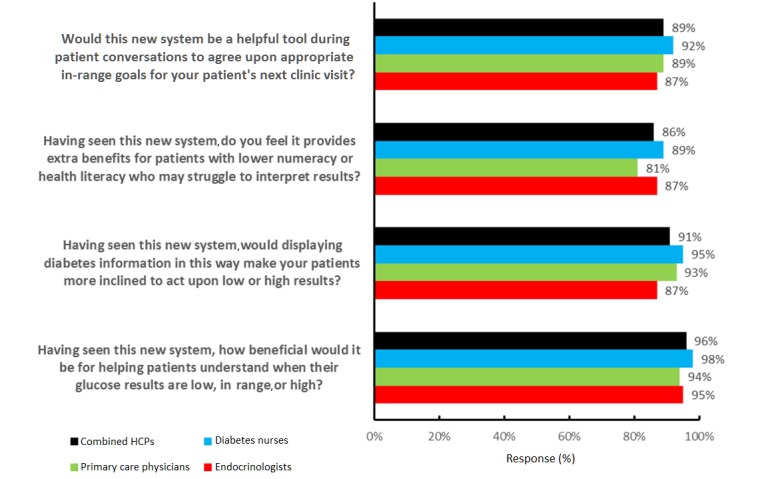
Health care professionals' (HCPs) clinical practice outlook after experiencing the OneTouch Verio Reflect meter and the OneTouch Reveal mobile app. Results shown are percentage favorable responses (strongly agree or agree; very beneficial or beneficial) on a 5-point Likert scale ranging from 1 (strongly agree or very beneficial) to 5 (strongly disagree or not at all beneficial). All percentage favorable responses met the acceptance criteria (ie, lower bound of 95% confidence limits >50%).

Having seen the new meter and app, 87.3% (124/142) of endocrinologists, 80.6% (87/108) of PCPs, and 88.6% (93/105) of nurses agreed it was beneficial for helping patients with lower numeracy or health literacy who may struggle to interpret results. In terms of supporting comprehension of glucose data, 95.1% (135/142) of endocrinologists, 94.4% (102/108) of PCPs, and 98.1% (103/105) of nurses agreed the system was beneficial for helping patients understand when their glucose results are low, in range, or high; 87.3% (124/142) of endocrinologists, 92.6% (100/108) of PCPs, and 95.2% (100/105) of nurses agreed that displaying diabetes information in this way would make their patients more inclined to act upon low or high results. With respect to improving patient consultations, 87.3% (124/142) of endocrinologists, 88.9% (96/108) of PCPs, and 92.4% (97/105) of nurses agreed this new system would be a helpful tool during patient conversations to agree upon appropriate in-range goals for their patients’ next clinic visit (see Figure 5).

## Discussion

This online study suggests that HCPs from seven countries had high overall acceptance of a new glucose meter and mobile app. This study also confirmed that using color-coded information and a BSM feature to assist patients with interpreting and acting upon their blood glucose information is an approach that resonates universally with different types of HCPs from different health care environments. We gathered feedback from a large sample of HCPs from each of seven countries and ensured that, collectively across all seven countries, we had a large number of endocrinologists, PCPs, and diabetes nurses to enable robust survey responses.

We found good agreement between the three types of HCPs in terms of the current self-care practices of patients and the issues that they face. HCPs were broadly very concerned that poor numeracy or health literacy were stumbling blocks that prevented their patients from being successful in aspects of diabetes care. Furthermore, it was interesting that HCPs felt that the majority of their patients had a good awareness about what represents a low, in-range, or high result; this viewpoint was expressed on behalf of patients with T1D and, to a marginally lesser extent, on behalf of patients with T2D. Surprisingly, given a more direct question specifically asking if the current glucose meters used by their patients made it easy for them to understand if their results were in range, agreement from HCPs of all types plummeted to between 55% and 65%. It is somewhat disappointing that after almost 30 years of companies developing home blood glucose meters, only around 60% of HCPs feel these devices provide this valuable context to patients, in parallel to their numerical result. Immediately alerting the patient to whether their on-screen result is in range, low, or high should be an expectation from the latest blood glucose meters. Interestingly, HCPs were skeptical that most of their T2D patients understood the reasons why they got low or high results, with less than 50% of HCPs agreeing that they did. Conversely, over 70% of HCPs agreed that T1D patients understood why they got low or high results, which is a higher percentage, although not a ringing endorsement and may be expected given that T1D patients test more frequently and routinely make therapy decisions based on results. In contrast to the HCP feedback in this study where between 86% and 96% of HCPs felt that their patients were aware of the context of their glucose results, our previous research with patients contradicts this feedback. We found that T1D and T2D patients struggle to categorize glucose results as low, in range, or high but did improve after experiencing a color range indicator feature explaining how to categorize different glucose readings [10]. Furthermore, our study showed that only 50% of HCPs felt T2D patients took action for low or high results; this low percentage mirrors the feedback obtained in a survey of insulin-using T2D patients in the United States who also responded that they would only take action for around 50% of their low or high glucose values [6]. Unsurprisingly, HCPs were more optimistic that T1D patients would take action for lows or highs, perhaps responding with more urgency based on the advice from the HCPs to avoid prolonged exposure to hypo- or hyperglycemia.

Technological advances now and in the future will present both opportunities and challenges. There is a real concern that HCPs may become overloaded with patient data from multiple devices (ie, continuous glucose monitors [CGMs], insulin pumps, health records, activity trackers, and health monitors), with an expectation that they analyze and translate copious amounts of information into actions for their patients [15]. Given that it remains the case that the average face-to-face time spent by a physician with a patient with diabetes is measured in mere minutes [16], it is essential to provide solutions that offer simple, automatic, data-driven advice directly to patients between consultations. Clearly, CGM companies are seeking to provide greater insight and decision support to patients between visits to improve glycemic control, although it must be acknowledged that these products remain relatively expensive and, for many patients, it may be worth first transitioning to more-advanced glucose meters. In this regard, HCPs in our online study experienced a BSM feature that resides directly on the meter and can communicate via Bluetooth to the Reveal mobile app, which provides additional BSM features. Our HCPs felt that the automatic insights and analysis of the glucose data provided by the BSM had important benefits in comparison to systems they use now, including automatically identifying times when patients are likely to experience hyper- and hypoglycemic events, analyzing patterns and tracking trends to help guide patients toward better self-management, and giving insights and encouragement to help patients take action. These are all areas to support patients between consultations to enable them to stick to the goals that have been set with HCPs during those infrequent office visits. Given the propensity for depression and anxiety among some diabetes patients, it was particularly important to ensure that the pattern and BSM messages conceived on our system were positive and routinely focused on getting back in range or achieving more in-range results rather than demotivating patients.

The most encouraging aspect of the surveys was how HCPs responded to statements about how the features and benefits of this new meter and app might contribute to the future care of their diverse patient populations. The diabetes community has been exploring new end points for diabetes care, going beyond HbA_1c_, and the idea of focusing on time or data-in-range has gained traction [17-19]. HCPs in our study agreed that our new meter and app would help them set appropriate in-range goals between visits, given that the meter clearly indicates, by pointing to a green bar, when results are in range. Facilitating this new and very practical way of ensuring patients maintain glycemic control is a key benefit. HCPs also acknowledged, prestudy, that they are concerned about basic numeracy and literacy and how it impacts their patients’ ability to self-manage. It was evident that the majority of HCPs felt the new meter and app could overcome some of these challenges to allow patients to better interpret and act upon their blood glucose data. It is worth noting some wider variations between HCPs for certain questions. For example, fewer endocrinologists (75%) agreed that the meter would help patients stay on top of their testing routine and control their glucose around meals, activities, and specific times of day, compared to 87% of PCPs and 82% of nurses. Similarly, fewer endocrinologists (73%) agreed the meter and app would allow them to quickly see and assess their patients’ lifestyle and blood glucose data, compared to 84% of PCPs and 86% of nurses. On the whole, PCPs and nurses were more aligned on their responses than endocrinologists, which may be partly explained by the more complex patients referred for secondary or specialty care.

In terms of study limitations, it is worth noting that these results may not be generalizable to HCPs in other countries that were not part of the survey and that despite recruiting a good sample of at least 50 HCPs per country, this is sample is only representative of the views of HCPs in each country. The plethora of color-enhanced features and displays now becoming routinely available from most manufacturers on glucose meters, diabetes apps, and sensor technologies (ie, CGMs) should be considered by HCPs with respect to those patients who may have some form of color visual impairment.

In conclusion, this multi-country online study provides evidence that HCPs were highly satisfied with the OneTouch Verio Reflect meter and OneTouch Reveal mobile app. Each of these use color-coded information and a BSM feature to assist patients with interpreting, analyzing, and acting upon their blood glucose results, which is particularly beneficial to keep patients on track during and between scheduled office visits.

## Abbreviations

BSM: Blood Sugar Mentor
CGM: continuous glucose monitor
FGM: flash glucose monitor
HbA_1c_: glycated hemoglobin
HCP: health care professional
PCP: primary care physician
T1D: type 1 diabetes
T2D: type 2 diabetes
